# The effect of *Bacopa monnieri* on gene expression levels in SH-SY5Y human neuroblastoma cells

**DOI:** 10.1371/journal.pone.0182984

**Published:** 2017-08-23

**Authors:** How-Wing Leung, Gabriel Foo, Gokulakrishna Banumurthy, Xiaoran Chai, Sujoy Ghosh, Tora Mitra-Ganguli, Antonius M. J. VanDongen

**Affiliations:** 1 Program in Neuroscience and Behavioral Disorders, Duke-NUS Medical School, Singapore, Singapore; 2 GlaxoSmithKline Consumer Healthcare, Gurgaon, Haryana, India; University of Oklahoma Health Sciences Center, UNITED STATES

## Abstract

*Bacopa monnieri* is a plant used as a nootropic in Ayurveda, a 5000-year-old system of traditional Indian medicine. Although both animal and clinical studies supported its role as a memory enhancer, the molecular and cellular mechanism underlying Bacopa’s nootropic action are not understood. In this study, we used deep sequencing (RNA-Seq) to identify the transcriptome changes upon Bacopa treatment on SH-SY5Y human neuroblastoma cells. We identified several genes whose expression levels were regulated by Bacopa. Biostatistical analysis of the RNA-Seq data identified biological pathways and molecular functions that were regulated by Bacopa, including regulation of mRNA translation and transmembrane transport, responses to oxidative stress and protein misfolding. Pathway analysis using the Ingenuity platform suggested that Bacopa may protect against brain damage and improve brain development. These newly identified molecular and cellular determinants may contribute to the nootropic action of Bacopa and open up a new direction of investigation into its mechanism of action.

## Introduction

*Bacopa monnieri* (Bacopa), also known as *Bacopa monniera*, *Herpestis monniera*, water hyssop or Brahmi, has long been used in Indian traditional medicine (Ayurveda) as a brain tonic to enhance memory performance, learning and concentration [1, 2]. These traditional claims have recently been supported by several animal and clinical studies. Animals treated chronically with Bacopa showed better acquisition and improved retention in learning tasks [3–11]. Similarly, clinical studies and a meta-analysis of randomized control trials also demonstrated that chronic oral administration of Bacopa (over a period of more than 12 weeks) to healthy subjects resulted in improvements in the subjects’ information processing speed, free recall, verbal memory and learning. Bacopa treatment also resulted in a decrease in anxiety, which improved learning [12–19]. With its low toxicity risks and apparent beneficial effects as a nootropic [20–22], Bacopa has been extracted and marketed as a dietary supplement (KeenMind or CDRI08, Soho Flordis International) and its production and imports are tightly inspected by the Food and Drug Administration (FDA) (https://tinyurl.com/ybkmhfes). Notwithstanding its wide availability, the mechanisms of action of Bacopa have yet to be delineated. Several mechanisms have been proposed concerning the nootropic effects of Bacopa. These included alteration of the levels of several neurotransmitters, including serotonin (5-hydroxytryptamine, 5-HT) [6, 23, 24], acetylcholine [4, 13, 25] and dopamine [6, 20, 24, 26]. Elevation of the neurotransmitter 5-HT resulted in activation of cAMP response element-binding protein (CREB) and subsequent changes in transcription, protein phosphorylation and histone modification [8, 23, 27]. Other research groups have suggested that Bacopa regulate pre- and post-synaptic proteins [9] and induce the formation of new dendrites [11, 28]. These proposed mechanisms are not mutually exclusive and the discrepancies could be attributed to a selective bias in the targets that were investigated by each research group.

In order to better define the molecular and cellular components of Bacopa action, we have applied a deep sequencing technique, RNA-Seq, to identify the changes in the transcriptome upon Bacopa treatment. We have performed both a transcript level and gene level analysis to investigate the changes in gene expression caused by Bacopa. These experiments have suggested underlying mechanisms of action for Bacopa, the biological pathways altered by this plant extract and the potential upstream mediators for these processes.

## Materials and methods

### Cell culture, differentiation of SH-SY5Y cells and Bacopa treatment

**Fig 1:**
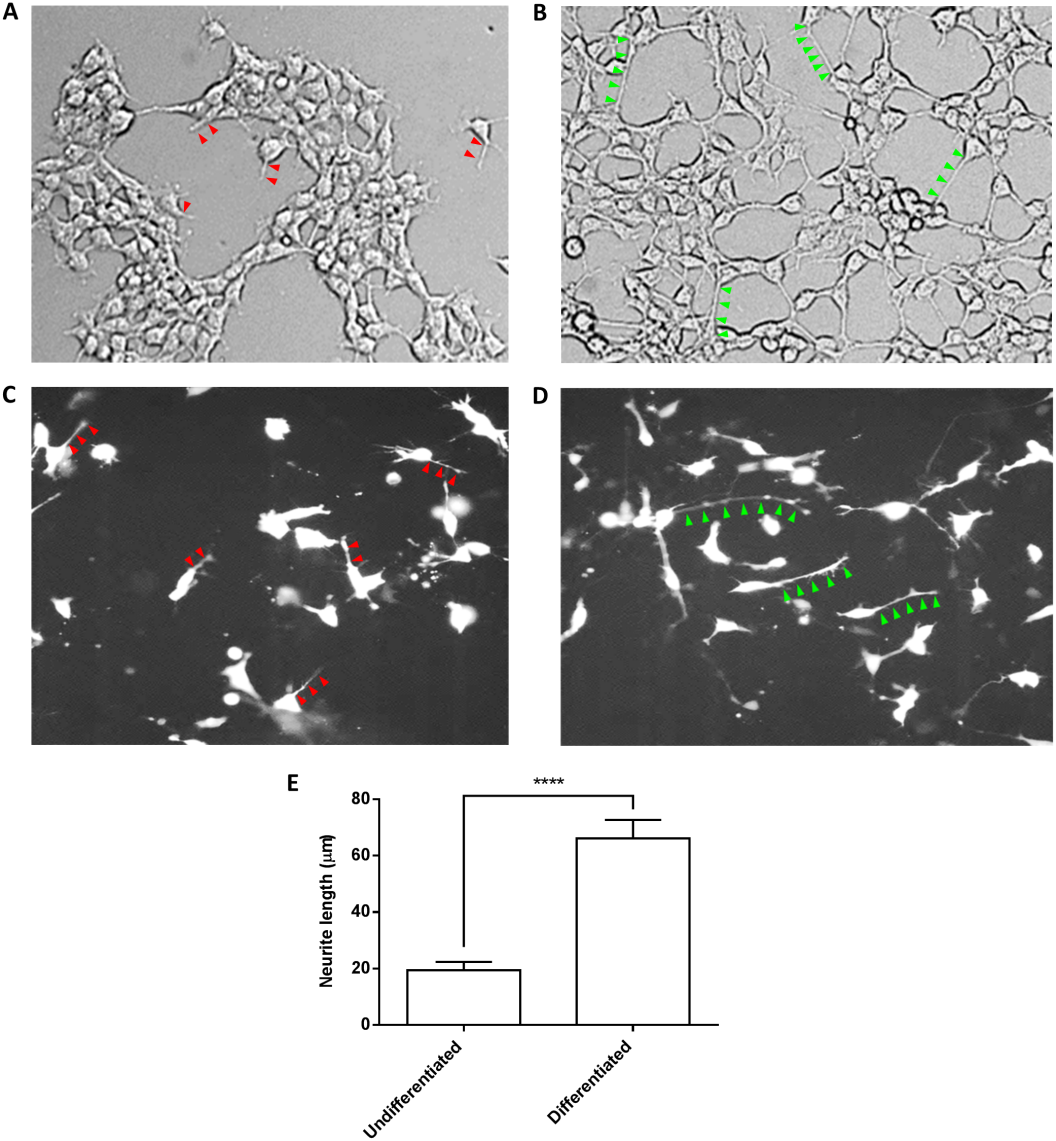
Differentiation of SH-SY5Y cells using laminin and IGF-1. SH-SY5Y cells were plated on laminin and grown for 24 hours in DMEM/F12 supplement and 10% FBS. To induce differentiation, FBS was removed and 50 nm IGF-1 was added; cells were allowed to grow for 72 hours. (A) Differential interference contrast (DIC) image of the undifferentiated controls. Red arrows marked the neurites in undifferentiated cells that were characteristic for neuroblast-like cells. (B) DIC image of the differentiated cells. The increase in neurite length upon differentiation was marked out by the green arrow heads. (C and D) To quantify the change in the length of the neurites, two days into the differentiation protocol, cells were transfected with GFP cDNA and imaged on day 3 using fluorescence microscopy. Transfecting with GFP highlighted the neurites among the confluent cell layers, allowing for easy quantification. (C) An overview of the undifferentiated controls. Red arrows marked out the neurite of each GFP transfected cells. (D) Differentiated cells displayed long neurites as outlined by green arrow heads. (E) The increase in the length of the neurites upon differentiation was statistically significant (unpaired t-test, **** indicates *P*-val < 0.0001).

The human neuroblastoma cell line, SH-SY5Y, was purchased from the American Type Culture Collection (ATCC CRL-2266). Cells were cultured in DMEM/F12 (Sigma) supplemented in 10% (v/v) fetal bovine serum (FBS, Gibco) and 1% (v/v) penicillin/streptomycin (P/S, JR Scientific). This medium will be referred to as *Complete Medium* henceforth. Subculturing was performed as per manufacturer’s instructions (ATCC). In brief, as the SH-SY5Y cells grow as a mixture of floating and adherent cells, care was taken to ensure the floating cells in the medium were collected and recovered by centrifugation. These collated floating cells would be combined with trypsinized adherent cells and subcultured. Cells were also passaged less than three times to ensure that the cells remained neuroblast-like [29] (Fig 1A and 1C). For experiments involving undifferentiated SH-SY5Y cells, the plating density was 0.4 x 10^6^ cells/cm^2^. To differentiate SH-SY5Y cells, they were plated at a density of 0.5 x 10^6^/cm^2^ on culture surfaces coated with 10 μg/ml laminin (Sigma) and maintained in *Complete Medium* for 18 h. After which, they were maintained in serum-free Complete Medium. 50 nM of human insulin-like growth factor-I (IGF-1) (Sigma) was added to promote differentiation [30]. 48 h after the switch to serum-free Complete Medium and the addition of IGF-1, the medium was replenished. Bacopa treatment was carried out 72 h after the start of differentiation. Undifferentiated and differentiated cells were treated with 3 μg/ml Bacopa for 24 h or 10 μg/ml Bacopa for 4 h, or with vehicle controls. For all experiments, we used a standardized extract of Bacopa (CDRI-08), containing no less than 55% bacoside A and bacoside B as its bioactive components that was extracted by ethanol extraction (Laila Impex, Vijaywada, India) [31, 32].

### Hydrogen peroxide (H_2_O_2_) toxicity assay

Undifferentiated or differentiated SH-SY5Y cells were exposed to different concentrations of H_2_O_2_ for 24 h. Number of live cells were measured using the LIVE/DEAD assay (Invitrogen) using high-content screening (HCS) microscopy platform (MetaXpress, Molecular devices). High (10 μg/ml) and low concentration (1 μg/ml) of Bacopa were supplemented during the addition of H_2_O_2_ for investigation of neuroprotective effects of Bacopa.

### RNA sample preparation, library construction and RNA sequencing

RNA was harvested from treated SH-SY5Y cells using the NucleoSpin RNA/Protein kit (Macherey-Nagel GmbH). Library construction and RNA sequencing were performed by the Duke-NUS Genome Biology Facility (DGBF). Briefly, 2.2 μg of RNA was sent for library construction. Quality of RNA was analyzed with the Agilent Bioanalyzer and RNA with RIN > 9 was used. Following poly-A enrichment, recovered RNA was processed using the Illumina TruSeq RNA Sample Preparation Kit v2 protocol (non-stranded) to generate adaptor-ligated libraries. A total of six samples were sequenced, obtained from undifferentiated and differentiated SH-SY5Y cells treated with (i) vehicle-treated control, (ii) 3 μg/ml Bacopa for 24 h, and (iii) 10 μg/ml Bacopa for 4 h. Samples were sequenced using two lanes of an Illumina HiSeq2000 sequencer using 76-bp paired-end reads.

### Computational analyses of RNA-sequencing data

RNA-Seq data was mapped to the human genome using Partek Flow (version 4.0.15.0406) and Partek Genomics Suite (version 6.15.0327). After the adaptor sequences were trimmed away, reads were mapped to the *Homo sapiens* genome (hg38) with TopHat2 (version 2.0.8). Local alignment was performed on the unaligned reads from TopHat2 to the human genome (hg38) with Bowtie2 (version 2.1.0). Aligned reads from the TopHat2 and Bowtie2 alignment were combined in Partek Flow. Post-alignment QA/QC was performed after each alignment step and aligned reads had an average quality Phred score above 30. The unique paired reads were used for gene expression quantification. Reads were assigned to individual transcripts of a gene based on the Expectation/Maximization (E/M) algorithm [33]. In the Partek Genomics Suite software, the E/M algorithm was modified to accept paired-end reads, junction aligned reads, and multiple aligned reads if these are present in the data. RNA expression was calculated as fragments per kilobase of transcript per million mapped reads (FPKM) values of the human RefSeq genes for paired-end sequencing. To identify differentially expressed genes, Partek’s Gene Specific Analysis (GSA) algorithm was used. Read counts between samples were normalized with the Upper Quantile method and analysis was performed at the transcript level. A cutoff value of multimodal P < 0.05 and fold change > 2 or < -2 were set. A gene ontology analysis was conducted using Partek Genomics Suite.

### Functional class scoring using gene-set enrichment and over-representation analysis

To identify biological functions affected by differential gene expression, evidence for functional class enrichment involving biological pathways or gene-sets was sought via two independent methods. First, pathway enrichment analysis was conducted via the Gene Set Enrichment Analysis tool using the “pre-ranked” option [34]. For this analysis, a total of 10744 genes with a FPKM ≥ 5 in at least 1 sample were included, and ranked by their fold-change in expression between the control and Bacopa treated samples. The ranked gene-list was used to query pathways from Gene Ontology-Biological Process and Gene Ontology-Molecular Function, (downloaded from the Molecular Signatures Database, MSigDB, [35], as well as custom pathways (Reactome pathways plus user-defined gene-sets for brain-specific functions) for statistically significant enrichment of higher or lower ranked genes. Significance estimates were adjusted for multiple testing via the false discovery rate (FDR).

Over-representation analysis (ORA) of biological functions and putative upstream regulators was carried out by subjecting a pre-filtered list of 576 differentially expressed genes (FPKM ≤ 5 in at least 1 sample and absolute fold-change ≥ 1.5) to QIAGEN’s Ingenuity Pathway Analysis tool (IPA, QIAGEN Redwood City, https://www.qiagenbioinformatics.com/product-login/). First, reference gene-sets corresponding to ‘biological functions’ (as defined in the Ingenuity Knowledge Base), were analyzed via Fisher’s exact test for statistically significant over-representation in the list of differentially expressed genes. Additionally, predictions of changes in the activity status of ‘upstream regulators’ (specifically, transcription factors), that could putatively explain the observed gene expression changes due to Bacopa treatments, was also carried out. An ORA was first performed to determine whether an upstream regulator was enriched for differential expression of its target genes (the list of regulators and their target genes were, again, obtained from the Ingenuity Knowledge Base). The overall activation or inhibition status of the regulator was then inferred from the degree of consistency (up- or down-regulation) in the expression patterns of its target genes, expressed as a z-score. Regulators with z ≥ 2 or z ≥ −2 were considered to be activated or inhibited, respectively.

### cDNA synthesis and quantitative Real-Time Polymerase Chain Reaction (qRT-PCR)

RNA was harvested using the NucleoSpin RNA/Protein kit (Macherey-Nagel GmbH). The amount of RNA was measured spectrophotometrically using a Nanodrop ND-1000 Spectrophotometer. cDNA was generated from 2 μg of RNA using the iScript cDNA synthesis kit (Bio-Rad Laboratories). Random hexanucleotides were annealed for 5 min at 25°C. cDNA synthesis was performed for 30 min at 42°C, followed by an enzyme inactivation step for 5 min at 85°C. cDNA was stored at -20°C until use. 1 μl of the cDNA reaction mix was used for qRT-PCR, which was performed using iQ SYBR green reagents (Bio-Rad Laboratories) on the iQ5 Multicolor Real-Time PCR Detection System (Bio-Rad Laboratories) with the following PCR profile: 95°C for 3 min, 40 cycles of 95°C for 10s and 55°C for 30 s. After the completion of the PCR, melt curve analysis was performed using the following paradigm: 95°C for 1 min, 55°C for 1 min followed by ramping up the temperature from 55°C to 95°C. RPL19 was used as a control.

## Results

### Characterization of SH-SY5Y cells

The goal of these experiments was to characterize the effects of Bacopa on functional properties and gene expression profiles of SH-SY5Y human neuroblastoma cells. In the presence of serum, undifferentiated SH-SY5Y cells can be propagated indefinitely, while they can be made to terminally differentiate by withdrawing serum, plating on a properly coated surface and addition of differentiating factors. SH-SY5Y cells (passage number 27, www.atcc.org) were differentiated by plating them on laminin-coated coverslips, using a protocol optimized by Dwane et al [30], which consisted of removing fetal bovine serum (FBS) and adding 50 nM Insulin Growth Factor 1 (IGF-1). Cells showed a differentiated neuronal phenotype (formation of extensive neurites) after 3–5 days (Fig 1B). In order to obtain a better visualization of the individual cells, we transfected the cultures with a cDNA encoding Green Fluorescent Protein (GFP) and used fluorescence microscopy to document the differentiation (Fig 1D and 1E). Fig 1 illustrates the results of differentiation on the morphology of SH-SY5Y cells. Upon differentiation, there was a significant 3.4 fold increase in the neurite length in the SH-SY5Y cells (undifferentiated cells: 19.4 μm; differentiated cells: 66.2 μm, student t-test, P-val = 0.0001) (Fig 1E).

### RNA-Seq analysis

In order to understand the changes in gene expression levels caused by Bacopa, we performed a comprehensive RNA-Seq analysis for both undifferentiated and differentiated SH-SY5Y neuroblastoma cells. Two different concentrations and treatment times were used: 10 μg/ml for 4 hours and 3 μg/ml for 24 hours. These concentrations were determined in pilot experiments to be the highest concentrations that were not deleterious to the cells. Vehicle (DMSO) was used as a control for the Bacopa effect. In addition, we evaluated the effect of differentiation itself. For each condition, the sequence of 50 million mRNA fragments (“reads”) was obtained which were then mapped to the human genome, as described in the Methods section, resulting in a relative abundance for all mRNAs expressed at a reasonable level. For each “treatment”, a list of differentially expressed mRNAs was generated, using a P-value smaller than 0.05 and an absolute fold-change of at least 2. Table 1 summarizes the results.

**Table 1:** Number of mRNAs altered by differentiation and Bacopa treatment.

Effect of	# mRNAs with fold change > 2	# neuronal mRNAs
Differentiation (laminin, IFG-1)	502	78
10 μg/ml Bacopa 4 hours (Undifferentiated)	57	1
3 μg/ml Bacopa 24 hours (Undifferentiated)	37	4
***10 μg/ml Bacopa 4 hours (Differentiated)***	***66***	***20***
3 μg/ml Bacopa 24 hours (Differentiated)	29	4

We considered mRNA levels to be altered if the P-value was smaller than 0.05 and the absolute value of the fold change was larger than 2. Four hours of Bacopa on differentiated cells was most effective (highlighted in bold and italics).

### Effect of differentiation

Differentiation of SH-SY5Y cells was accomplished by growing them on laminin and replacing serum with IGF-1. Cells showed a differentiated phenotype (formation of extensive neurites) after 3–5 days (Fig 1B, 1D and 1E). The RNA-Seq analysis detected 31,500 different transcripts in SH-SY5Y cells. Due to alternative splicing, the number of distinct mRNAs in a cell is typically larger than the number of genes (~23,000) in the human genome. The differentiation protocol resulted in a change of 502 mRNA levels, of which 78 could be classified as transcribed from ‘neuronal’ genes (S1 Table). The results indicated that (1) our differentiation protocol was effective in altering the gene expression profile towards a more neuronal phenotype, and (2) the RNA-Seq approach can be used to identify changes in mRNA levels in an un-biased manner and at a genome wide scale.

### Effect of Bacopa treatment: Individual transcript level analysis

**Fig 2:**
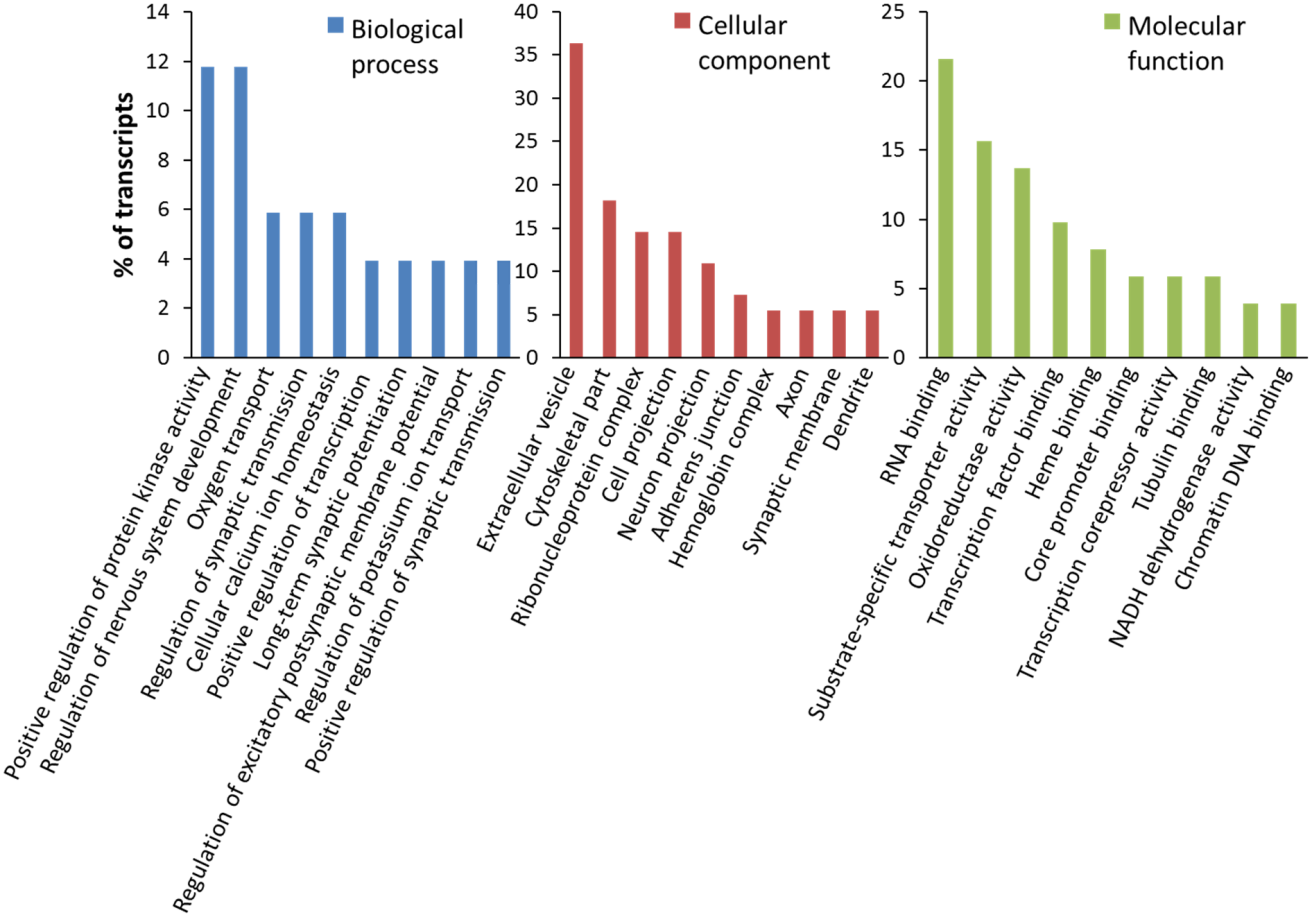
Results of the gene ontology analysis by Partek Genomics Suite. The bar graphs indicate the percentage of transcripts that belong to Biological Processes (blue), Cellular Components (red) and Molecular Functions (green) that were most affected by Bacopa treatment.

Table 1 summarizes the effect of four different Bacopa treatment protocols (2 concentrations/durations in undifferentiated and differentiated SH-SY5Y cells) on the mRNA profile of the neuroblastoma cells. The 4-hour treatment with Bacopa altered the expression of more mRNAs than the 24-hour Bacopa treatment: 57 and 66 vs. 37 and 29 altered mRNAs in undifferentiated and differentiated cells, respectively (Table 1). This indicated that the effect of Bacopa on gene transcription seen after 4 hours subsides after one day for many of the affected transcripts. 4 hours of Bacopa on differentiated cells was most effective, with 66 mRNAs being altered at least 2-fold (Table 1). This data set was therefore selected for further analysis. A gene ontology analysis was conducted using Partek Genomics Suite. Fig 2 illustrates the results, listing the biological processes, cellular components and molecular functions affected by Bacopa treatment. Many of the affected biological processes referred to functions that were of critical importance in the central nervous system. Table 2 summarizes the mRNAs altered by Bacopa treatment encoded by genes with a neuronal function. The most striking finding was the Neuroplastin gene (NPTN), because a single nucleotide polymorphism (SNP) in the Neuroplastin locus associates with cortical thickness and intellectual ability in adolescents [36]. Neuroplastin is a synaptic glycoprotein involved in long-term potentiation (LTP) in hippocampal CA1 synapses that modulates neuritogenesis and neuronal plasticity [37, 38].

**Table 2:** Summary of neuronal genes whose mRNAs were altered by Bacopa treatment of SH-SY5Y cells.

Gene Symbol	Gene name	Fold change	Function
HNRNPC	Heterogeneous nuclear ribonucleoprotein C	19.7	Promotes APP translation by competing with FMRP for APP mRNA recruitment to P bodies
CIB2	Calcium and integrin binding family member 2	6.7	Role in Ca^2+^ homeostasis and Ca^2+^ regulation in the mechano-transduction process; Mutations cause deafness
NPTN	Neuroplastin	6.6	Regulation of long-term neuronal synaptic plasticity, cytosolic Ca^2+^ ion concentration, neuron projection development; SNPs associated with cognitive abilities in adolescents
COMMD6	COMM Domain Containing 6	6.4	NF-KappaB binding; Regulates transcription factor activity, gene expression
NDUFA5	NADH Dehydrogenase 1 Alpha Subcomplex 5	(4.7)	Mitochondrial transport
CHAC1	ChaC Glutathione-Specific Gamma-Glutamylcyclotransferase 1	4.0	Negative regulator of Notch signaling pathway involved in embryonic neurogenesis; Promotes neurogenesis in embryos
AP2S1	Adaptor-Related Protein Complex 2, Sigma 1 Subunit	3.8	Synaptic transmission; Regulates EGFR, TRK receptor, ephrin receptor pathway
KCNMA1	K Channel, Ca Activated Large Conductance M Alpha 1	(3.3)	Regulation of membrane potential; Synaptic transmission
NGFR	Nerve Growth Factor Receptor	3	Mediates cell survival and cell death of neural cells; Necessary for circadian oscillation in suprachiasmatic nucleus
STRN3	Striatin-3	2.6	Glutamate regulation of dopamine D1A receptor signaling
PRKACB	Protein Kinase, CAMP-Dependent, Catalytic, Beta	(2.6)	Mediates signaling through cAMP; Involved in neuronal structure and signaling
LDHA	Lactate Dehydrogenase A	(2.5)	Substantia nigra development
MTMR2	Myotubularin Related Protein 2	2.3	Mutations result in Charcot-Marie Tooth disease type 4B, an autosomal recessive demyelinating neuropathy
VCL	Vinculin	2.3	Involved in regulation of actin cytoskeleton, axon and neuron projection extension; Has a negative regulation on cell migration
WDR1	WD Repeat Domain 1	2.3	Involved in sensory perception of sound, regulation of oligodendrocyte differentiation or gliogenesis and neurogenesis
DBI	Diazepam Binding Inhibitor (GABA Receptor Modulator, Acyl-CoA Binding Protein)	(2.3)	Modulates signal transduction at GABA_A_ receptors; Displaces diazepam from the benzodiazepine recognition site in GABA_A_ receptor
EFNB2	Ephrin-B2	(2.3)	Mediate development of the nervous system; Crucial for migration, repulsion and adhesion during neuronal development
ACTB	Actin, Beta	2.2	Involved in axonogenesis, axon guidance, neuron projection morphogenesis, substantia nigra development, ATP-dependent chromatin remodeling, ephrin receptor signaling pathway
ACTG1	Actin Gamma 1	2.2	Associated with DFNA2-/26, a subtype of autosomal dominant non-syndromic sensorineural progressive hearing loss; Involved in Ras signaling pathway, axonal guidance
SLC38A1	Solute Carrier Family 38, Member 1	2.2	Glutamine transporter, precursor for the synaptic transmitter, glutamate and GABA; Involved in synaptic transmission and neurotransmitter reuptake
STMN4	Stathmin-Like 4	(2.2)	Involved in neuron projection development, microtubule depolymerization, neuronal plasticity, rapidly induced after seizure or LTP
CALR	Calreticulin	2.1	Major Ca^2+^-binding protein in the lumen of the ER; Essential for integrin-mediated signaling and cell adhesion
SLC1A4	Solute Carrier Family 1 (Glutamate/Neutral Amino Acid Transporter), Member 4	2.1	Associated with Hartnup disorder; Chloride channel activity; Transporter for alanine, serine, cysteine and threonine, sodium dependent
KLHL24	Kelch-Like Family Member 24	(2.1)	Reduces kainate receptor-mediated currents in hippocampal neurons
ATF4	Activating Transcription Factor 4	2	Transcriptional activator; Protects against neuronal death in Parkinson's disease; Involved in neurodegeneration; May constrain long-term synaptic changes and memory formation
NRCAM	Neuronal Cell Adhesion Molecule	(2.0)	Involved in neuron-neuron adhesion; Promotes directional signaling during axonal cone growth
STMN1	Stathmin 1	(2.0)	Required for axon formation during neurogenesis; Involved in the control of learned and innate fear
XRCC6	X-Ray Repair Complementing Defective Repair in Chinese Hamster Cells 6	(2.0)	Involved in brain development; Positive regulation of neurogenesis

### Effect of Bacopa treatment: Gene level analysis

The analysis described above was based on mapping the reads to individual transcripts of the human genome (see Materials and methods). Because of the extensive alternative splicing seen for many genes, a process that is most prominent in the brain, mapping the reads is a difficult process and bound to be error-prone. We have therefore used an additional analysis, which is more robust, in which the reads mapped to all exons belonging to a gene were combined to provide a gene level statistic. Several additional genes regulated by Bacopa were identified this way, as summarized in Table 3.

**Table 3:** Genes regulated by Bacopa identified by gene-level analysis of the RNA-Seq data.

Gene Symbol	Gene name	Fold Change	Function
HBA2	Hemoglobin, Alpha 2	405	Oxygen-transport metalloprotein in the red blood cells
HBB	Hemoglobin, Beta	370	Oxygen-transport metalloprotein in the red blood cells
HBA1	Hemoglobin, Alpha 1	292	Oxygen-transport metalloprotein in the red blood cells
ANKRD1	Ankyrin Repeat Domain 1	21.7	Transcription factor involved in development and under conditions of stress
SLC7A11	Solute Carrier Family 7 Member 11	7.5	Transporter that antiports glutamate for cysteine
SERPINE1	Serpin Peptidase Inhibitor, Clade E	6.8	Serine protease inhibitor that functions as the principal inhibitor of tissue plasminogen activator and urokinase
WNT8B	Wingless-Type MMTV Integration Site Family, Member 8B	6.8	Wnt isoform specific for the developing brain
HIST1H4K	Histone Cluster 1, H4k	5.3	Histone H4 isoform
HIST1H4J	Histone Cluster 1, H4j	5.2	Histone H4 isoform
CCL2	Chemokine (C-C Motif) Ligand 2	4.8	Recruits monocytes, memory T cells, and dendritic cells to sites of inflammation produced by injury or infection
CHAC1	ChaC Glutathione-Specific Gamma-Glutamylcyclotransferase 1	4.5	Proapoptotic component of the unfolded protein response; Downstream of the ATF4-ATF3-CHOP cascade
NTS	Neurotensin	4.4	Neuropeptide implicated in the regulation of hormone release; Has interaction with the dopaminergic system
ANGPTL4	Angiopoietin-Like Protein 4	4.0	Induced under hypoxic conditions; Serum hormone directly involved in regulating lipid metabolism
PTPRH	Protein Tyrosine Phosphatase, Receptor Type, H	(3.8)	Ubiquitously expressed; Upregulated in gastrointestinal cancers
TXNIP	Thioredoxin Interacting Protein	(3.8)	Glucocorticoid-regulated primary response gene involved in mediating glucocorticoid-induced apoptosis
YPEL4	Yippee-Like 4 (Drosophila)	(3.6)	Nuclear protein; Activates Elk-1 in the MAPK signaling pathway; Possible function in cell division
CNN2	Calponin 2	3.5	Actin-binding protein implicated in cytoskeletal organization
RAPGEF4	Rap guanine nucleotide exchange factor 4	(3.0)	EPAC2; May regulate synaptic plasticity
STON1	Stonin 1	(3.0)	Component of the endocytic machinery
FMN1	Formin 1	(3.0)	Role in the formation of adherens junction and the polymerization of linear actin cables

### Validation of the RNA-sequencing results: qRT-PCR

**Fig 3:**
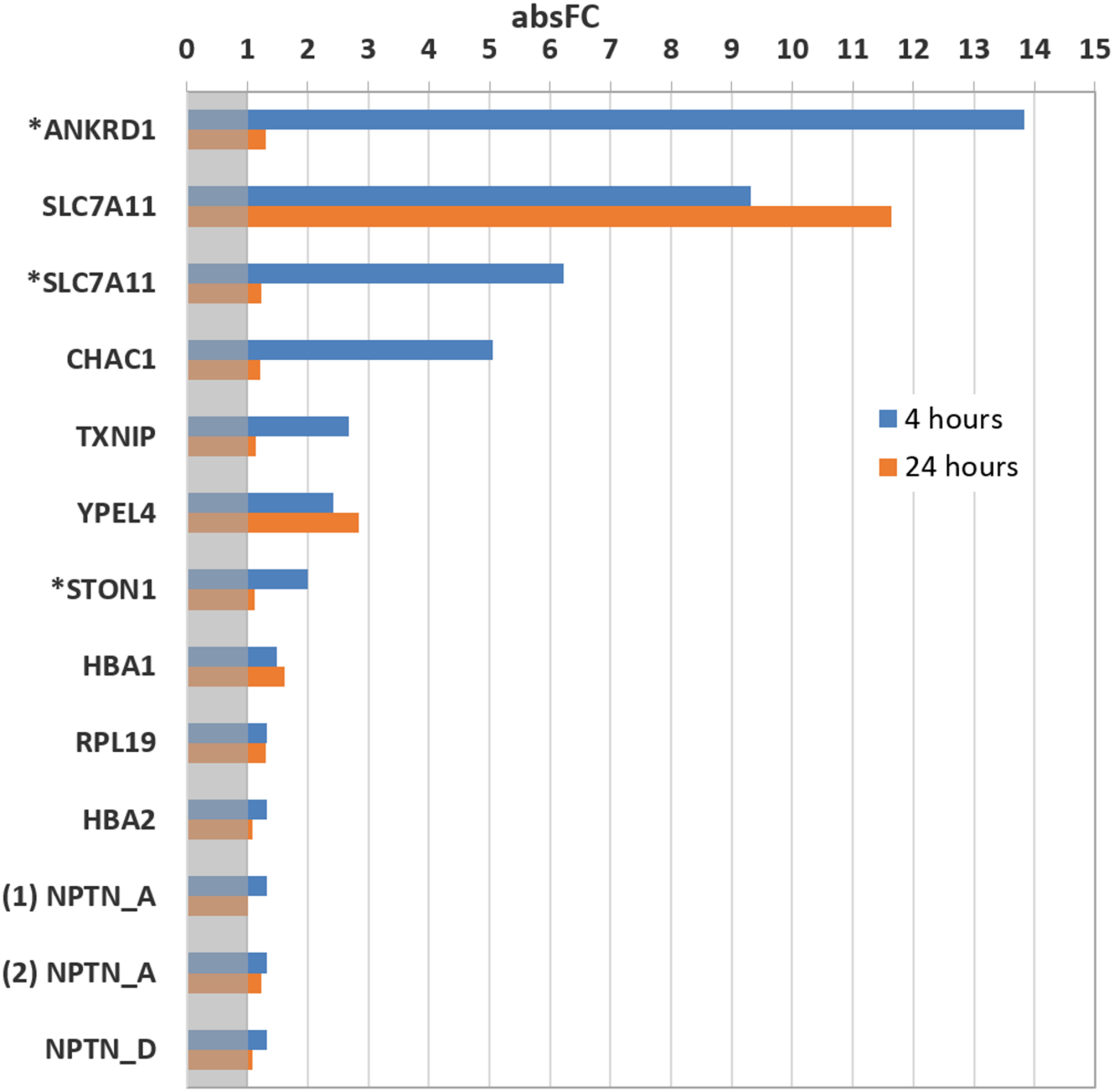
Absolute values of Fold Change (absFC) caused by Bacopa treatment. RT-PCR was performed on undifferentiated cells and differentiated cells which were treated with vehicle (DMSO) or with Bacopa for either 4 h (blue) or 24 h (orange). The gray area indicated an absFC value smaller than 1. Genes marked with * were results from the treatment on undifferentiated cells. (1) NPTN_A and (2) NPTN_A were results generated from 2 sets of primers priming for NPTN transcript A.

Next, we validated the RNA-Sequencing results using qRT-PCR experiments for a subset of genes. Fig 3 summarizes the results by showing the absolute value of the fold change produced by Bacopa treatment. In the case of genes with alternatively spliced transcripts, PCR primer pairs were designed that were unique for the mRNA isoform that was altered by Bacopa. In some cases, this proved to be hard, since the exon that was unique for the altered transcript was very short. A case in point is Neuroplastin, which has four mRNA variants, one of which is affected by Bacopa (NPTN_D). The distinguishing feature of the affected variant is the absence of exon 2 and that exon 7 is shortened by 12 nucleotides. We were able to validate the effects of Bacopa on the transcripts identified by the gene-level analysis (ANKRD1, SLC7A11, CHAC1, TXNIP, YPEL4 and STON1) (Table 3). However, for the mRNAs for which only one or a few of the alternatively spliced variants were affected by Bacopa, the validation was less successful: the effects were either much smaller than those seen in the RNA-Seq analysis (HBA1 and HBA2) (Table 3) or there was no measurable effect (NPTN_D) (Table 2).

### Gene Set Enrichment Analysis (GSEA)

**Fig 4:**
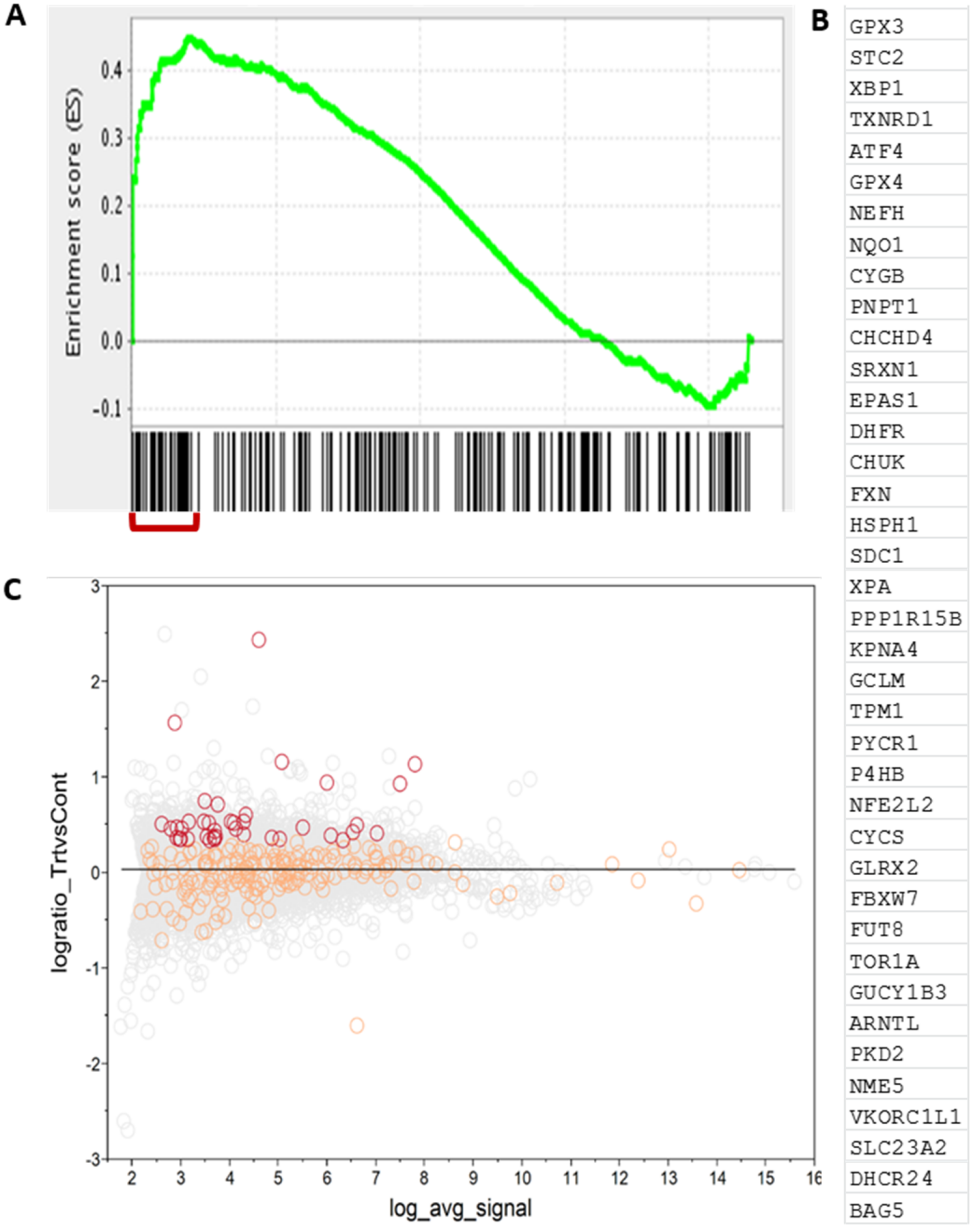
Enrichment Plot, MA Plot and enriched gene list for the ‘oxidative stress response’ pathway. (A) Enrichment Plot for the designated pathway. The graph represented the incremental change in the enrichment score for this pathway, when queried along the ranked list of genes during gene-set enrichment analysis (GSEA). Maximal enrichment score was observed at 0.45. The relative ranks of the genes belonging to this pathway were indicated by the bars under the graph. Lines clustered to the left (marked in red) demonstrated strong enrichment of 39 highly ranked genes for this pathway that were upregulated by Bacopa. (B) The 39 genes (leading up to the maximal enrichment score) that contributed positively to the core enrichment of the ‘oxidative stress response’ pathway in GSEA. (C) Mean-Average (MA) plot analysis of the Oxidative Stress Response pathway. The MA plot compares the distribution of differential gene expression as a function of the magnitude of expression signals. The Y-axis plotted the log ratio of treatment (Bacopa) vs. Control (DMSO) and X-axis recorded the log of the average FPKM score between the two groups. The distribution of log ratios for all genes queried by RNA sequencing were shown in light gray circles. Genes belonging to the Oxidative Stress Response pathway (238 genes) were shown in orange and red circles. The 39 genes contributing to significant enrichment of this pathway (by GSEA) were shown in red whereas the remaining pathway genes were shown in orange.

The deep sequencing experiments (RNA-Seq) we have performed yielded a set of genes that were differentially affected by Bacopa treatment. In order to better understand the underlying biological processes affected by Bacopa, we compared the genes affected by Bacopa to the gene sets belonging to each of the entries in the Gene Ontology database. A statistical test was performed for each GO term to investigate if it is enriched for the Bacopa-regulated genes. The Broad Institute has developed a set of tools to query the Molecular Signatures Database (MSigDB), a collection of annotated gene sets. Table 4 shows the results of a GSEA of three databases: Reactome, Gene Ontology (Molecular Function: GOMF, Biological Process: GOBP) and Canonical. Twenty-five gene sets were identified with a significant enrichment score (P-val < 0.05). Twenty-two of these fell into three broad functional categories: (1) oxidative stress response, (2) translation regulation, and (3) membrane transport. Fig 4 provide more detailed results for the ‘Oxidative Stress Response’ gene set, including the Enrichment plot (Fig 4A), the genes enriched in this pathway (Fig 4B) and the Mean-Average (MA) plot (Fig 4C).

**Table 4:** Results from GSEA (Pre-ranked).

Name Pathway	Function	Size	NES	P-val	Dir	Database
Antioxidant activity	Oxidative stress	11	1.86	0.038	Up	GO
Activation of chaperones by ATF6 alpha	Unfolded protein response	11	1.78	0.053	Up	Reactome
Cytosolic tRNA aminoacylation	Oxidative stress	22	1.97	0.011	Up	Reactome
Oxidative stress response	Oxidative stress	238	1.44	0.036	Up	Custom
Influenza viral RNA transcription and replication	Translation regulation	101	-1.52	0.05	Down	Canonical
KEGG_ribosome	Translation regulation	82	-1.86	0.004	Down	Canonical
Peptide chain elongation	Translation regulation	86	-1.89	0.004	Down	Reactome
3'UTR mediated translational regulation	Translation regulation	106	-1.55	0.043	Down	Reactome
Structural constituent of ribosome	Translation regulation	74	-1.72	0.015	Down	GO
tRNA aminoacylation	Translation regulation	36	-1.74	0.044	Up	Reactome
Amino acid transport across the plasma membrane	Transmembrane transport	10	1.86	0.017	Up	Reactome
amino acid and oligopeptide SLC transporters	Transmembrane transport	14	1.92	0.033	Up	Reactome
SLC mediated transmembrane transport	Transmembrane transport	96	1.56	0.038	Up	Reactome
Transmembrane transporter activity	Transmembrane transport	10	1.78	0.051	Up	GO
Ligase activity forming carbon oxygen bonds	Metabolism	12	1.88	0.027	Up	GO
Acetylglucosaminyltransferase activity	Metabolism	11	-1.77	0.053	Down	GO

Three different databases were used from the MSigDB at the Broad Institute: ‘Reactome’ (674 gene sets), Gene Ontology’ (GO, 396 gene sets), and ‘Canonical’ (1330 gene sets). **Size** = number of genes in the set, **NES** = Normalized Enrichment Score, **Dir** = direction. Only results with P-val < 0.055 are listed. The identified gene sets could be assigned to one of four biological functions: stress response (blue), translation regulation (grey), membrane transport (yellow) and metabolism (green). ATF6: Activating transcription factor 6; 3’UTR: 3’ Untranslated region; SLC: solute-carrier.

### Ingenuity Pathway Analysis (IPA)

With the current results, we were interested to understand the possible upstream biological causes and potential downstream effects. As such, we performed IPA on the RNA-Seq data for Bacopa treatment of differentiated SH-SY5Y cells. IPA infers statistically, based on the altered gene expression in our dataset, the possible downstream effects on biological functions and association with diseases (Table 5) and Upstream Regulators (Table 6). Table 5 shows four Biological Functions identified by IPA that were over-represented with differentially expressed genes in Bacopa-treated vs. Control samples: (1) carbohydrate metabolism, (2) generation of cells, (3) migration of cells, and (4) cell proliferation. Six Biological Functions were predicted to be inhibited by Bacopa treatment, including (1) organismal death, (2) damage of brain, (3) apoptosis and (4) synthesis of reactive oxygen species (ROS), and (5) growth failure. Note that the activation states of these disease-related categories were decreased by Bacopa treatment. Many of these Bacopa effects would therefore be expected to contribute to increased brain health and improved brain development. Table 6 shows the three Upstream Regulators (transcription factors) that the IPA analysis predicted as differentially regulated based on the observed effects of Bacopa treatment on gene transcription. The transcription factors, Activating Transcription Factor 4 (ATF4) and Nuclear Factor, Erythroid 2 Like 2 (NFE2L2) were predicted to be activated, while Forkhead Box O3 (FOXO3) was inhibited. ATF4 is a transcription factor involved in the endoplasmic reticulum (ER) stress response [39]. It is known to collaborate with NFE2L2 (aka NRF2), a master redox switch that turns on cellular signaling involved in the induction of cytoprotective genes [40–43]. FOXO3 is a ‘forkhead box’ transcription factor that regulates autophagy and neural oxidative stress-mediated stem cell homeostasis and has been associated with longevity in worms [44–48]. Therefore, all three upstream regulators identified by IPA are transcription factors involved in regulating cellular stress pathways.

**Table 5:** Ingenuity Pathway Analysis: Over-represented biological functions.

Categories	Diseases or function Annotation	P-val	Predicted activation state
Carbohydrate metabolism	Quantity of carbohydrate	6.01E-05	Increased
Cellular growth and Proliferation; Tissue development	Generation of cells	1.34E-04	Increased
Cellular movement	Migration of stem cells	2.01E-04	Increased
Cellular Development; Cellular growth and proliferation	Cell proliferation of breast cancer cell lines	3.38E-04	Increased
Organismal survival	Organismal death	6.14E-06	Decreased
Neurological disease; Organismal injury and abnormalities	Damage of brain	1.73E-05	Decreased
Cell death and survival; Neurological disease	Apoptosis of cerebral cortex cells	6.09E-04	Decreased
Cell death and survival; Neurological disease	Apoptosis of cortical neurons	9.93E-04	Decreased
Free radical scavenging	Synthesis of reactive oxygen species	1.26E-03	Decreased
Developmental disorder	Growth failure	6.61E-03	Decreased

**Table 6:** Ingenuity Pathway Analysis: Upstream regulators.

Upstream	Exponential	Molecular type	Predicted	Activation	Bias-corrected	P-val of
regulator	log ratio		activation state	z-score	z-score	Overlap
FOXO3	n.s.	Transcription	Inhibited	-2.772	-2.841	4.53E-05
		regulator				
NFE2L2	n.s.	Transcription	Activated	2.722	2.579	4.18E-04
		regulator				
ATF4	0.937	Transcription	Activated	3.404	3.218	2.62E-19
		regulator				

IPA identified three transcription factors that each regulated the transcription of genes whose mRNA levels were affected by Bacopa treatment. This suggests that Bacopa exert its effect by inhibiting FOXO3 and activating both NFE2L2 (NRF2) and ATF4 (CREB2). The transcription level of FOXO3 and NFE2L2 was not significantly (n.s.) changed by Bacopa, but they were predicted to be functionally inhibited and activated, respectively, by the treatment, based on the observed transcriptional response of their target genes. ATF4, in contrast, was also transcriptionally regulated by Bacopa (exp. Log ratio = 0.937).

### Neuroprotection

**Fig 5:**
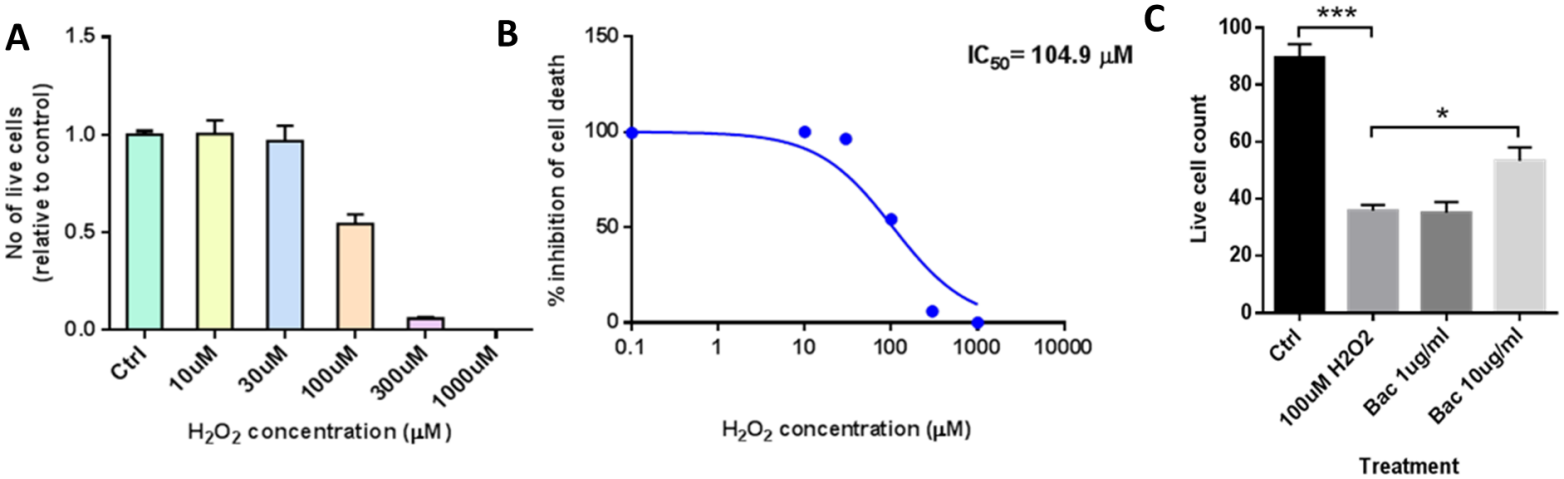
H_2_O_2_ toxicity and protection by Bacopa. (A) Using a high-content screening (HCS) microscopy platform (MetaXpress, Molecular Devices) we measured the number of live SH-SY5Y cells after a 24-hour treatment with H_2_O_2_ at the concentrations indicated. The number of live cells per field of view was normalized to the control value (B) H_2_O_2_ survival curve fitted with the logistics equation: The IC_50_ (concentration that kills 50% of the cells) was 105 μM. (C) Neuroprotection assay for Bacopa. The bar graphs represented live cell counts per field of view (means ± SEM) for (i) control cultures (Ctrl), (ii) cells treated with 100 μM peroxide for 24 hours, and cells treated with the same amount of H_2_O_2_ supplemented with a (iii) high and (iv) low concentration of Bacopa (Bac). The high concentration Bacopa showed significant protection, while the low concentration failed to protect against peroxide toxicity. * P-val < 0.05, *** P-val < 0.001, one-way ANOVA, Dunnett post-test.

To validate the findings obtained from the RNA-Seq data, we have performed a functional oxidative stress assay of Bacopa and studied the neuroprotective properties of Bacopa. SH-SY5Y cells were first challenged with hydrogen peroxide (H_2_O_2_) and the effect was evaluated using a fluorescence-based live-death assay. Fig 5A and 5B illustrate the concentration-survival curve for H_2_O_2_. H_2_O_2_ was quite efficacious in causing cell death. Bacopa was able to partially protect against H_2_O_2_ toxicity, at a concentration of 10 μg/ml, while the lower dose of 1 μg/ml showed no efficacy in this neuroprotection assay (Fig 5C). Protection was seen at a lower dose than previously reported for Bacopa in SH-SY5Y cells, in which the lowest dose tested was 25 μg/ml [49]. This neuroprotective property of Bacopa seen in these functional oxidative stress assays validated the results and analysis obtained from the RNA-Seq, which suggested an effect of Bacopa on the oxidative stress response pathway.

## Discussion

By identifying changes in the gene transcription profile in human neuroblastoma cells (SH-SY5Y) using an RNA-Seq approach, we have identified some of the molecular/cellular components and pathways that underlie the mechanism of action of *Bacopa Monnieri* (Bacopa). Changes in mRNA levels produced by Bacopa treatment were validated using qRT-PCR. A gene ontology analysis of the mRNAs affected by Bacopa indicated effects on brain development, Ca^2+^ and K^+^ ion homeostasis, synaptic function and long-term potentiation (Fig 2), providing the first mechanistic support for the well-published nootropic effects of Bacopa on cognitive function and memory performance.

The Gene Set Enrichment Analysis (GSEA) performed for the effect of 4 hours of Bacopa treatment on differentiated SH-SY5Y neuroblastoma cells identified three biological functions/pathways affected by Bacopa: 1) oxidative stress response, 2) transmembrane transport and 3) translation regulation (Table 4). The first two pathways were upregulated by Bacopa and the third was downregulated, suggesting an inhibition of general translation. Interestingly, aminoacylation of tRNAs was upregulated by Bacopa, which would indicate that the levels of tRNAs will increase, thereby stimulating translation rates. It is possible that the overall effect of Bacopa treatment is an introduction of a selective bias in the translation of a specific class of mRNAs. This would have to be investigated further.

A second interesting biological function uncovered by the GSEA was the up-regulation by Bacopa of a select group of members of the SLC family of solute carriers, which consists of over 300 proteins functionally grouped into 52 sub-families, including facilitative transporters, primary and secondary active transporters, ion channels, and the aquaporins. Bacopa increased the expression of 31 (out of 300) SLC family members. Table 7 summarizes the function of some of these transmembrane carriers by listing the molecules they transport. Many have critical functions in the central nervous system. For instance, Glutamate, L-DOPA, norepinephrine and monoamines are neurotransmitters, or their precursors. The Na^+^/Ca^2+^ exchanger and K-Cl co-transporter are critical for intracellular ion homeostasis and neuronal excitability. Zinc is a co-factor that regulates NMDA receptor function. Finally, there are transporters for glucose, fatty acids, phosphate and many amino acids, which are required for normal metabolism. These transporters are heteromeric complexes assembled from multiple subunits. Bacopa altered the mRNA levels for two and four subunits for the complexes that transport glucose and Zinc respectively.

**Table 7:** Summary of SLC-mediated transport functions up-regulated by Bacopa.

Gene	Transports
SLC33A1	Acetyl-CoA
SLC27A4	Fatty acids
SLC2A1	Glucose
SLC2A8	Glucose
SLC1A4	Glutamate
SLC38A1	Glutamine
SLC7A5	L-DOPA; Amino acids (W, Y, L, R, F)
SLC8A3	Na^+^/Ca^2+^-exchanger
SLC12A5	Neuronal K-Cl cotransporter
SLC6A2	Norepinephrine
SLC20A1	Phosphate
SLCO4A1	Thyroid hormones T3 and T4
SLC35A2	UDP-Galactose
SLC35A3	UDP-N-Acetylglucosamine
SLC18A1	Monoamines (vesicular)
SLC30A1	Zinc
SLC30A5	Zinc
SLC39A3	Zinc
SLC39A6	Zinc

Moreover, Bacopa was found to upregulate many genes that respond to oxidative stress, thereby likely improving the capacity of the cell to properly deal with such insults. Oxidative damage resulted from reactive oxygen species caused pathological manifestations of aging resulting in cognitive dysfunction [50]. Overexpression of superoxide dismutase in aged mice exhibited enhanced hippocampal LTP, better cerebellum-dependent motor learning and better hippocampus-dependent spatial learning [51]. Similarly, rats exposed to another antioxidant, Curcumin, also had improved memory retention [52]. Identification of the exact subset of genes of the oxidative stress response pathway that are controlled by Bacopa (Fig 4) now enables more mechanistic studies of the neuroprotective effect of Bacopa. This important finding goes a long way towards understanding the mechanism underlying Bacopa’s neuroprotection capabilities that others and we have characterized (Fig 5) [24, 25, 53–58].

**Fig 6:**
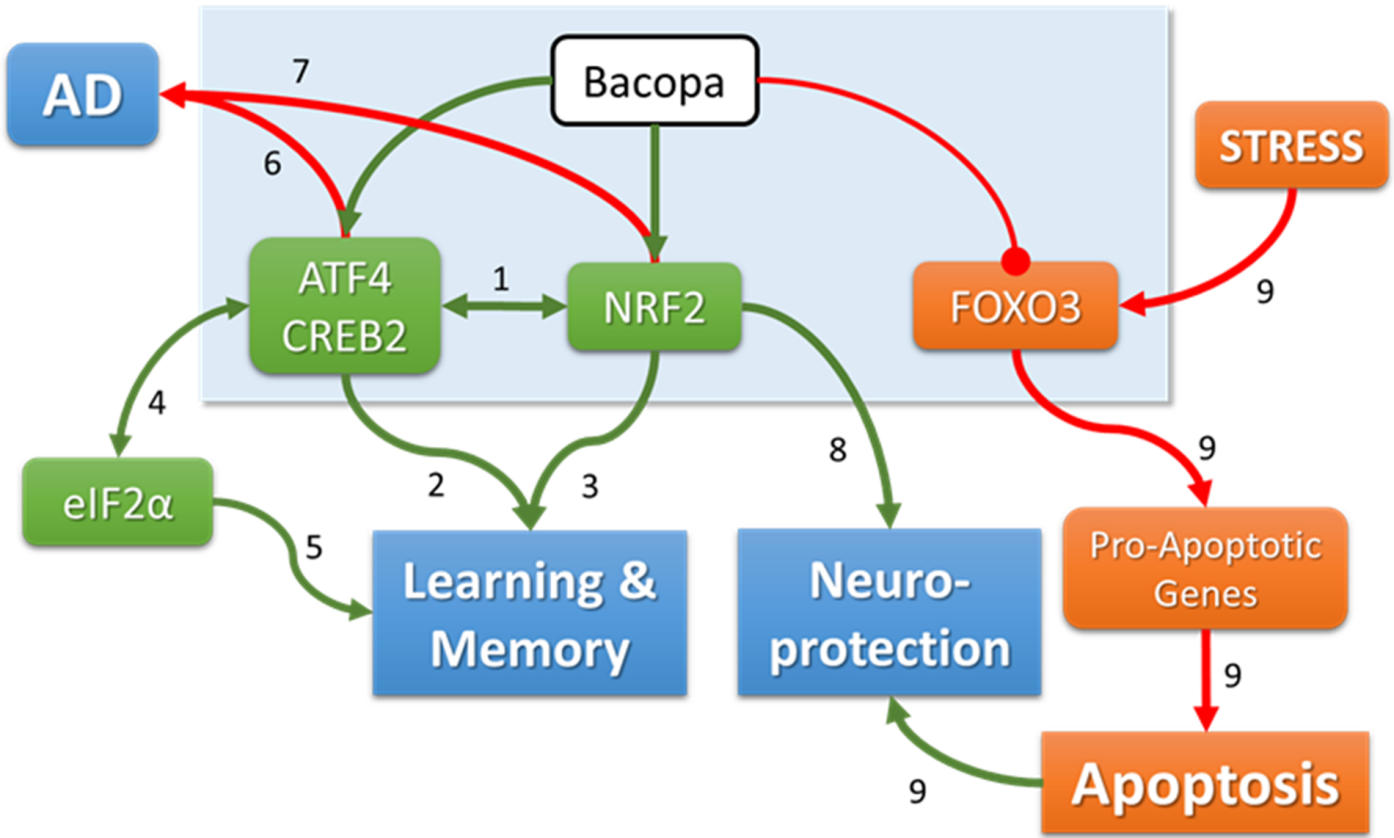
Pathways identified by IPA connects Bacopa to memory, neuroprotection and AD. The light-blue box summarizes the effect of Bacopa on the three transcription factors identified by the Ingenuity Pathway Analysis (IPA). Bacopa activates ATF4 and NRF2, while it inhibits the function of FOXO3. The color of boxes and arrows indicates the effects of Bacopa: green indicates increase/activate, orange indicates decrease/inhibit. Blue boxed are three biological endpoints: learning & memory, neuroprotection, and Alzheimer’s disease (AD). Explanation of the numbered connections: (1) ATF4 and NRF2 functionally interact with each other [40–43]. (2) ATF4 is implicated in memory [59–62]. (3) NRF is implicated in memory [63–67]. (4) Translation initiation factor eIF2-alpha stimulates translation of ATF4 [68–71]. (5) eIF2-alpha is implicated in memory formation [72, 73]. (6) ATF4 has been implicated in Alzheimer’s disease (AD) [74–78]. (7) NRF2 has also been linked to AD [63, 65, 67, 79]. (8) NRF2 plays a critical role in neuroprotection [80–83]. (9) FOXO3 mediates oxidative stress-induced neuronal cell death [84–87]. Inhibition of FOXO3 by Bacopa could explain its neuroprotective effect.

In addition, the ingenuity pathway analysis (IPA) identified biological functions that were increased by Bacopa such as cell proliferation and migration; and several neuronal disease-associated pathways, which were inhibited by Bacopa such as including brain damage, growth failure, apoptosis of neurons and oxidative stress damage (Table 5). The IPA analysis also identified three transcription factors which are likely responsible for the changes in gene expression seen following Bacopa treatment: ATF4 (CREB2), NFE2L2 (NRF2) and FOXO3 (Table 6). Taken together, the data outlined a specific set of biological pathways altered by Bacopa as well as the molecular players that mediated these effects. Fig 6 summarizes a working model showing how Bacopa could exert its effects on biological endpoints, neuroprotection and processes underlying learning & memory and Alzheimer’s disease (AD), by modulating the function of the identified transcription factors. The link to AD we discovered was unexpected, but there are a few publications suggesting protective effects of Bacopa in AD models [53, 58]. Further clinical studies of Bacopa’s therapeutic value in AD seem warranted.

In conclusion, the RNA-seq analysis presented here provides a new framework of transcription factors and the genes they control that may mediate the nootropic and neuroprotective effects of Bacopa.

## Supporting information

S1 Table List of neuronal mRNAs that were differentially regulated by Bacopa. (XLSX)
